# The Effect of Chelation on Blood Pressure in Lead-Exposed Children: A Randomized Study

**DOI:** 10.1289/ehp.8634

**Published:** 2006-01-24

**Authors:** Aimin Chen, George G. Rhoads, Bo Cai, Mikhail Salganik, Walter J. Rogan

**Affiliations:** 1 Epidemiology Branch, National Institute of Environmental Health Sciences, National Institutes of Health, U.S. Department of Health and Human Services, Research Triangle Park, North Carolina, USA; 2 Environmental and Occupational Health Sciences Institute, University of Medicine and Dentistry of New Jersey, Piscataway, New Jersey, USA; 3 Biostatistics Branch, National Institute of Environmental Health Sciences, National Institutes of Health, U.S. Department of Health and Human Services, Research Triangle Park, North Carolina, USA; 4 Harvard School of Public Health, Harvard University, Boston, Massachusetts, USA

**Keywords:** blood pressure, chelation, child, lead, succimer

## Abstract

Studies in children suggest a weak association between blood lead concentration and blood pressure. To understand this better, we tested the strength of the association in children with elevated blood lead concentrations and whether succimer chelation changed blood pressure as it did blood lead. In a randomized clinical trial of 780 children with blood lead concentrations of 20–44 μg/dL at 12–33 months of age, we compared the systolic and diastolic blood pressure in the succimer-treated group and placebo group for up to 5 years of follow-up. We also analyzed the relation of blood lead to blood pressure. Children in the succimer group had lower blood lead concentrations for 9–10 months during and after treatment, but their blood pressure did not differ from those in the placebo group during this period. During 1–5 years of follow-up, children in the succimer group had systolic blood pressure 1.09 (95% confidence interval, 0.27–1.90) mmHg higher than did untreated children in a model with repeated measurements, but the difference in diastolic blood pressure was not statistically significant. No association between blood lead and blood pressure was found. Overall, there is no association between blood lead and blood pressure in these children with moderately high lead exposure, nor does chelation with succimer change blood pressure.

Although the causal nature of the relation between lead exposure and elevated blood pressure in adults is still debated [Agency for Toxic Substances and Disease Registry (ATSDR) 1999; Den Hond et al. 2002, 2003; Vupputuri et al. 2003], a meta-analysis of about 60,000 subjects from 31 studies estimated that a doubling of blood lead concentration was associated with a 1.0 mmHg [95% confidence interval (CI), 0.5 to 1.4 mmHg] rise in systolic pressure and a 0.6 mmHg (95% CI, 0.4 to 0.8 mmHg) rise in diastolic pressure (Nawrot et al. 2002). Children generally have higher blood lead concentrations, but data on the relation between blood lead concentration and blood pressure in them are sparse and were not included in the meta-analysis because “blood pressure in children is highly variable and correlates highly with height” (Nawrot et al. 2002).

One 1970s study showed that children with blood lead concentrations > 40 μg/dL had higher systolic (but not diastolic) blood pressure (Jhaveri et al. 1979), but other studies did not detect any positive association between blood lead and blood pressure in children < 10 years of age (Friedlander et al. 1981; Rogan et al. 1978; Selbst et al. 1993). These early studies all had < 150 subjects and did not adequately control confounding. A contemporary study of 282 children 5.5 years of age in Kosovo, in the former Yugoslavia, did adjust for several covariates with multiple regression (Factor-Litvak et al. 1996). That study showed that a 10-μg/dL increase in blood lead concentration was associated with a 0.5-mmHg (95% CI, −0.2 to 1.3 mmHg) increase in systolic and a 0.4-mmHg (95% CI, −0.1 to 0.9 mmHg) increase in diastolic blood pressure (Factor-Litvak et al. 1996). The authors claim that these results suggest but do not demonstrate a weak association (Factor-Litvak et al. 1996).

Blood lead concentration peaks at about 2 years of age and then declines, whereas blood pressure increases with age. It is plausible that the relation between blood lead and blood pressure differs in childhood and adulthood, or that it is unstable in childhood, and thus difficult to characterize with one measurement. Repeated measurements of blood lead and blood pressure in the same child might then be informative. In addition, if lead does increase blood pressure and the effect is acute or subacute and reversible, then the relation will be more apparent when body lead burden changes, such as during chelation.

We used data from a large, randomized study of chelation therapy for lead exposure to answer three questions: Does a sudden and substantial decrease in blood lead induced by chelation have any effect on blood pressure? Is a sustained but modest lowering of blood lead over a 6–9 month period associated with a change in blood pressure? And is there any association between concurrent blood lead and blood pressure over 5 years of follow-up in young children with significant, but variable, lead exposures?

## Materials and Methods

The Treatment of Lead-Exposed Children (TLC) trial is a randomized, placebo-controlled, double-blind clinical trial of 780 children 12–33 months of age (mean age, 2 years) with moderately high blood lead concentrations (20–44 μg/dL) to test the effect of chelation on cognitive function and behavior (TLC 1998). Four clinical centers, in Baltimore, Maryland; Cincinnati, Ohio; Newark, New Jersey; and Philadelphia, Pennsylvania, participated in the enrollment, treatment, and follow-up. Three hundred ninety-six children were treated with succimer (Chemet; McNeil Laboratories, Fort Washington, PA), an oral lead chelator, for up to three 26-day rounds (75% of children received a second round of drug, and 81% of those receiving a second round of treatment received a third); 384 were given placebo for similar periods. The study was approved by the institutional review boards at the clinical centers, Harvard School of Public Health, Centers for Disease Control and Prevention, and National Institute of Environmental Health Sciences. The parents of all the children provided written informed consent at enrollment. Succimer decreased blood lead level for several months but had no effect on IQ or neurobehavioral test scores at 36 months (about 5 years of age) and 60 months (about 7 years of age) after initiation of treatment (Dietrich et al. 2004; Rogan et al. 2001).

### Blood lead concentration measurements.

Blood lead concentrations were measured at baseline and days 7, 28, and 42 after the beginning of each round of treatment. Blood lead concentrations were also measured at 3- to 4-month intervals for 5 years of follow-up. The Nutritional Biochemistry Branch at the Centers for Disease Control and Prevention in Atlanta did the blood lead analyses by atomic absorption spectrometry based on the methods described by Miller et al. (1987).

### Blood pressure measurements.

At each visit for blood lead measurement, study nurses also measured systolic and diastolic blood pressures. A Dinamap Vital Signs Monitor (an automatic device; Critikon, Inc., Tampa, FL) was used for all blood pressure measurements. Blood pressure was measured when children were seated. The average of up to three measurements per visit (without a notation that the child was crying or not staying still) was used for statistical analysis. The overall average number of blood pressure measurements per visit was 2.2. At 36- and 60-month follow-ups, three blood pressure measurements were acquired from all subjects. Study nurses were blinded to treatment status of children.

### Statistical analysis.

We first tested the hypothesis that succimer lowered blood pressure by comparing the succimer and placebo groups immediately after initiation of treatment and at the subsequent follow-ups. We used multiple regression models of blood pressure by treatment groups to obtain the adjusted difference in blood pressures between treatment and placebo groups at each visit (baseline, during treatment, and at follow-up). Covariates adjusted for included clinical center, baseline blood lead concentration, race (black, nonblack), sex, parents’ education (< 12 years, 12 years, ≥13 years), single parent (yes, no), age at blood pressure measurement (in years), height, and body mass index (BMI) at measurements (Factor-Litvak et al. 1996; Sinaiko 1996). Mixed models with similar covariate adjustment were used to obtain adjusted regression estimates accounting for repeated measurements of blood pressure (using repeated statement in the modeling). The effects of baseline blood lead concentration and age at blood pressure measurement were modeled as fixed effects. Unstructured covariance structure was used in the mixed models by Akaike’s information criterion (Akaike 1974). Because the difference in blood lead concentrations between the succimer and placebo groups lasted 9–10 months after initiation of treatment, the interval from initiation of treatment to 9-month follow-up (not including baseline) and the interval from 12- to 60-month follow-up were modeled separately.

The trial data set is also a large observational cohort data set, so we also wanted to examine the relation between blood lead concentration and blood pressure in these data. To do this, we tested whether concurrent blood lead concentration was associated with blood pressure at various time points adjusted for treatment group and other covariates (clinic centers, race, sex, parents’ education, single parent, age at blood pressure measurement, height, and BMI). Scatter plots of blood pressure by blood lead concentration and plots of residual versus predicted values of regression models supported a linear relation between blood lead concentration and blood pressure, so linear regression models were used. We also did mixed models with repeated blood lead and blood pressure measurements to test their association for two above-mentioned intervals separately.

We used software R (version 2.0; R Development Core Team 2004) for smooth curve fitting and graphics; and SAS (version 9.0; SAS Institute Inc. Cary, NC) for general statistics, multiple regression models, and mixed models.

## Results

Of 780 enrollees, 44% were female, 77% were African American, 72% lived with a single parent, and 40% had parents with < 12 years of education. At baseline (mean ± SD), their height was 85.7 ± 5.8 cm, BMI was 16.7 ± 1.8 kg/m^2^, and age at blood lead and blood pressure measurement was 2.0 ± 0.5 years. No differences in these baseline characteristics were seen between treatment groups.

At baseline, the mean blood lead concentration of 780 children was 26 ± 5 μg/dL; there was no difference between the succimer and placebo groups in blood lead concentration at baseline. After initiation of treatment, children in the succimer group had lower blood leads than did those in the placebo group for about 9–10 months. The two groups then had similar blood lead concentrations until the end of study at 60-month follow-up.

**Figure 1:**
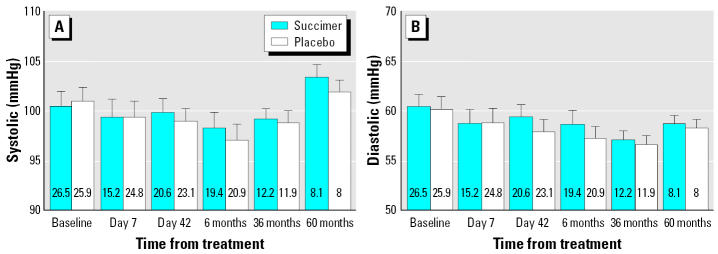
Systolic (*A*) and diastolic (*B*) blood pressure by treatment group at baseline, days 7 and 42 of first round of treatment, and 6-, 36-, and 60-month follow-ups in TLC trial children. Error bars represent upper bound of 95% CI of the mean blood pressure. Numbers within bars are blood lead concentrations (μg/dL) of each group at these time points.

Over the 5 years starting from randomization, the children’s blood lead concentrations declined by 70% (at 60-month follow-up, the mean blood lead concentration was 8 ± 4 μg/dL, but blood pressure changed little. Of 704 children (both succimer and placebo groups combined) who had blood pressure measurement at baseline, the mean systolic blood pressure was 100.7 ± 13.5 mmHg and the mean diastolic blood pressure was 60.3 ± 11.3 mmHg. No difference of blood pressure between succimer and placebo groups at baseline was observed. Figure 1 shows the mean systolic and diastolic blood pressure by treatment group at baseline, days 7 and 42 of the first round of treatment, and at 6-, 36-, and 60-month follow-ups. Although the blood lead concentration declined sharply in the succimer group at day 7 of the first round of treatment, blood pressure in the succimer group did not differ from that in the placebo group. Also, there was no difference in blood pressure between the two treatment groups at other time points. The results of regression models estimating differences in blood pressure between treatment groups (succimer vs. placebo) at different time points are shown in Table 1. For simplicity, results at only baseline, first round of treatment, and 6-, 12-, 18-, 24-, 36-, and 60-month follow-ups (when more children were measured) are presented. There were slight, statistically significant differences between succimer and placebo groups in systolic blood pressure at the 36- and 60-month follow-up visits, but only after adjustment for covariates. In these multiple regression models, baseline blood lead level was generally not associated with later blood pressure, whereas height and BMI were usually positively associated with blood pressure. Mixed models with adjustment of covariates showed that, for the interval from initiation of treatment to 9-month follow-up, the overall difference between the succimer and placebo groups is 0.24 mmHg (95% CI, −0.79 to 1.28 mmHg) for systolic blood pressure and 0.46 mmHg (95% CI, −0.44 to 1.36 mmHg) for diastolic blood pressure (both *p* > 0.05). Adjusted mixed models for the interval from 12-month to 60-month follow-up indicated a higher systolic blood pressure for the succimer group, with an estimated difference of 1.09 mmHg (95% CI, 0.27–1.90 mmHg; *p* < 0.05); the estimated difference in diastolic blood pressure is 0.15 mmHg (95% CI, −0.45 to 0.75 mmHg; *p* > 0.05).

**Figure 2:**
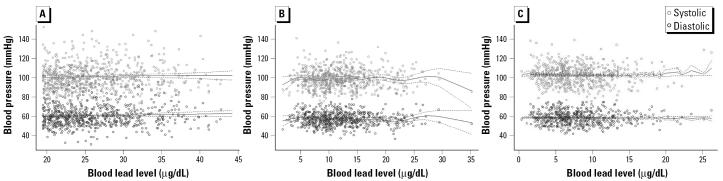
Scatter plots of systolic and diastolic blood pressure by concurrent blood lead concentration at baseline (*A*) and 36-month (*B*) and 60-month (*C*) follow-up. Solid lines are spline regression estimates without covariate adjustment, and dashed lines are their 95% CIs.

The cross-sectional association of blood lead levels and blood pressure were first plotted with data from baseline and 36- and 60-month follow-ups (Figure 2). Spline regression predictions are also shown. There was no obvious association between blood lead level and blood pressures at these age points. The parametric regression estimates from baseline, first round of treatment, and 6-, 12-, 18-, 24-, 36-, and 60-month follow-ups are shown in Table 2. At day 7 of the first round of treatment, blood lead concentration was associated with systolic blood pressure in an unadjusted analysis, but the association became nonsignificant after adjustment for covariates. Taking into account repeated measurements of each individual and adjusting for covariates, a mixed model using the interval between initiation of treatment and 9-month follow-up showed regression estimates of −0.16 mmHg (95% CI, −0.69 to 0.38 mmHg) in systolic blood pressure and 0.19 mmHg (95% CI, −0.27 to 0.66 mmHg) in diastolic blood pressure per 10-μg/dL blood lead increase. Using intervals from 12- to 60-month follow-up, the estimates of per 10-μg/dL blood lead increase were 0.01 mmHg (95% CI, −0.57 to 0.58 mmHg) in systolic blood pressure and 0.003 mmHg (95% CI, −0.45 to 0.45 mmHg) in diastolic blood pressure. None of these estimates in both intervals was statistically significant (all *p* > 0.05).

## Discussion

Using data from a large clinical trial of children with moderately high lead exposure, we found that succimer treatment did not change blood pressure from initiation of treatment through 9 months after treatment, although it decreased blood lead. In the interval from 1 year after treatment to 5 years after treatment, however, children in the succimer group had a 1-mmHg increase in systolic blood pressure compared with children given placebo; there was no difference in diastolic blood pressure. Analysis of most visits and mixed models considering repeated measurements did not show any consistent association between concurrent blood lead levels and blood pressures in children 2–7 years of age. Overall, the results suggest no association between blood lead and blood pressure in young children.

Lead exposure in experimental animals induces hypertension (Chai and Webb 1988; Ding et al. 1998; Preuss et al. 1994; Staessen et al. 1994; Victery 1988; Victery et al. 1982b). The possible mechanisms are intracellular perturbation of calcium metabolism at end arterioles and effects on the renin–angiotensin system (Sharp et al. 1987). The lead and blood pressure association has been seen in occupationally exposed workers (de Kort et al. 1987; Telisman et al. 2004), the general adult population (Cheng et al. 2001; Vupputuri et al. 2003), pregnant women (Rothenberg et al. 2002), and peri-menopausal women (Nash et al. 2003), but the literature is not entirely consistent (ATSDR 1999; Hertz-Picciotto and Croft 1993). In children, our study does not support an association between blood lead and blood pressure. The TLC trial children at enrollment had a mean blood lead concentration of 26 μg/dL, and it could be that the exposure level of lead is not high enough to produce effect on blood pressure. In the Kosovo study, children from the lead-exposed town had mean blood lead levels of 37 μg/dL, whereas children from the unexposed town had that of 9 μg/dL, but the blood pressure did not differ by town of residence. Also, when data from two towns were combined, the relation between blood lead and blood pressure was not statistically significant (Factor-Litvak et al. 1996). A much higher blood lead concentration (range, 40–90 μg/dL), as seen in the Jhaveri et al. (1979) study of children 1–3 years of age, may be detrimental to blood pressure, but our study could not test this hypothesis. In animal studies, *in utero* and postnatal exposure to 100 ppm lead in drinking water increased blood pressure, but exposure to 5 or 25 ppm did not, although renin activity was increased to 25 ppm (Victery et al. 1982a, 1982b). The 100-ppm dose group resulted in a blood lead concentration of 40 μg/dL, whereas the 25-ppm dose group had an average of 18 μg/dL. The hypertension effect seen in the high-dose group may be secondary to the renal lesion caused by continuous high exposure rather than being a direct effect (Victery 1988). Another possible reason for the null association observed in our study is that children with moderate lead exposure may not show elevated blood pressure until a later age, but we do not have data after 7–8 years of age. A previous report of 50-year follow-up of children with plumbism found a higher risk for hypertension in adulthood compared with controls, suggesting a very long induction period (Hu 1991).

It is also possible that we failed to detect an existing relation between blood lead and blood pressure. Although we have the largest sample size so far in published studies of blood lead and blood pressure in children (Factor-Litvak et al. 1996; Friedlander et al. 1981; Jhaveri et al. 1979; Rogan et al. 1978; Selbst et al. 1993), we only have power of 0.8 to detect a 2-mmHg difference in blood pressure between treatment and placebo groups for a given cross-sectional comparison. Similarly, for the regression model of blood pressure by blood lead, cross-sectional analysis at each time point was underpowered to detect a shallow slope. We did use repeated measurements of blood lead and blood pressure analyzed with a mixed model in an attempt to mitigate this concern. Even so, in our study, we may not have enough power to detect a slope of 0.5 mmHg per 10 μg/dL blood lead level as seen in the Yugoslavia study (Factor-Litvak et al. 1996). Use of an automated blood pressure monitor in our study is another concern. Although it is not the clinical standard, it can be used as a screening tool or in epidemiologic study of children (Gillman and Cook 1995; National High Blood Pressure Education Program 2004). The Yugoslavia study also used an automated monitor for blood pressure measurements (Factor-Litvak et al. 1996).

The TLC trial was designed to test the hypothesis that children with moderate blood lead levels receiving succimer treatment would have better scores on tests of cognition and behavior than children on placebo. Because no difference in cognitive and behavioral scores was observed between succimer and placebo groups (Dietrich et al. 2004; Rogan et al. 2001), we examined blood pressure, an interesting but not primary outcome in the trial. The finding of no change in blood pressure raises the questions whether the length and dose of treatment were enough to induce changes, whether new exposure to lead was effectively prevented, or whether the lead effects on blood pressure, if they exist, are likely to be reversible. In this trial, we used higher doses for a longer time than on the Chemet (succimer) label, and it is unlikely other chelation regimens are more effective (Rogan et al. 2001). However, even with this dose, only a small portion of total lead body burden was removed because most lead burden is in bone for both children and adults. A longer treatment length (more than three rounds) may reduce blood lead concentration for a longer time (> 10 months), but such treatments tend to be less feasible. The houses of TLC trial children were cleaned up by vacuuming, mopping, minor repairing, and paint stabilization to minimize continuous lead exposure (Rogan et al. 2001). Study in adults indicated that long-term lead accumulation might increase blood pressure and risk of hypertension (Cheng et al. 2001). We found succimer increased systolic blood pressure from 1 year to 5 years after treatment, but the difference was very small and might have been caused by something besides lead, such as calcium excretion from the treatment (Graziano et al. 1988). Calcium intake has been reported to be inversely associated with blood pressure in children (Gillman et al. 1992, 1995). It may also be that despite adjustment for covariates, residual confounding (e.g., genetics, dietary intake) still exists and contributes to the small difference in systolic blood pressure (Sinaiko 1996). Given these uncertainties, a logical step to study the association between lead exposure and blood pressure in children is to seek direct evidence of such an association, preferably by meta-analysis or pooled analysis.

Despite the limitations, this study had larger sample size and repeated measurements and thus more power and precision than previous studies in children. Common confounders such as age, height, and BMI were adjusted for in the analysis. The proportion of children retained in the TLC study is high, and those who remained in the follow-up did not differ in treatment group, race, sex, and socioeconomic status from those lost to follow-up.

The TLC trial children had much higher blood lead levels than the mean of 2 μg/dL in general U.S. children (Pirkle et al. 1998). It is unlikely that background exposure will have effects if the blood lead concentrations in the TLC trial did not. Although there is no clear evidence that the moderate lead exposure experienced by these children affected their blood pressure, the associations between lead exposure and decreased IQ in children and increased blood pressure in adults render it an important contributor to the global burden of disease (Fewtrell et al. 2004), hardly diminished by these relatively minor negative findings.

## Figures and Tables

**Table 1:** Regression estimates [β(95% CI)] of blood pressure by treatment groups (succimer vs. placebo) in TLC trial children.

		Systolic (mmHg)		Diastolic (mmHg)	
Visit	No.	Unadjusted	Adjusted^a^	Unadjusted	Adjusted^a^
Baseline	704	−0.54 (−2.54 to 1.47)	−0.80 (−2.27 to 1.17)	0.31 (−1.37 to 1.98)	0.15 (−1.50 to 1.80)
Treatment					
Day 7 of 1st round	509	0.05 (−2.23 to 2.33)	−0.37 (−2.58 to 1.84)	−0.14 (−2.04 to 1.75)	−0.86 (−2.70 to 0.99)
Day 28 of 1st round	429	0.05 (−2.37 to 2.47)	0.13 (−2.27 to 2.53)	0.77 (−1.33 to 2.87)	0.55 (−1.53 to 2.63)
Day 42 of 1st round	631	0.81 (−1.04 to 2.67)	0.54 (−1.24 to 2.32)	1.54 (−0.09 to 3.16)	1.16 (−0.43 to 2.76)
Follow-up					
6 months	365	1.19 (−1.04 to 3.43)	1.10 (−1.02 to 3.21)	1.44 (−0.37 to 3.24)	1.32 (−0.48 to 3.11)
12 months	597	0.87 (−0.77 to 2.51)	0.87 (−0.66 to 2.40)	0.90 (−0.31 to 2.12)	0.87 (−0.36 to 2.09)
18 months	335	0.76 (−1.23 to 2.75)	0.89 (−0.95 to 2.73)	–0.48 (−1.94 to 0.98)	−0.44 (−1.88 to 1.01)
24 months	607	0.40 (−1.10 to 1.90)	0.64 (−0.66 to 1.94)	−0.81 (−1.88 to 0.25)	−0.79 (−1.81 to 0.23)
36 months	647	0.43 (−1.08 to 1.95)	1.27 (0.06 to 2.48)*	0.54 (−0.49 to 1.57)	0.74 (−0.26 to 1.74)
60 months	569	1.57 (−0.09 to 3.22)	1.69 (0.34 to 3.04)*	0.36 (−0.78 to 1.50)	0.30 (−0.80 to 1.39)

Estimates are estimated differences between succimer and placebo children.

a Adjusted for clinical center, baseline blood lead level, race, sex, parents’ education, single parent, age at test, height, and BMI.

* *p* < 0.05.

**Table 2:** Regression estimates [β(95% CI)] of blood pressure change per 10 μg/dL of elevation in concurrent blood lead level in TLC trial children.

	Systolic (mmHg)		Diastolic (mmHg)	
Visit	Unadjusted	Adjusted^a^	Unadjusted	Adjusted^a^
Baseline	1.19 (−0.76 to 3.15)	1.36 (−0.58 to 3.30)	1.25 (−0.38 to 2.88)	1.47 (−0.16 to 3.10)
Treatment				
Day 7 of 1st round	1.41 (0.13 to 2.69)*	1.26 (−0.27 to 2.78)	1.05 (−0.01 to 2.12)	1.14 (−0.13 to 2.41)
Day 28 of 1st round	0.02 (−1.50 to 1.53)	0.21 (−1.53 to 1.95)	0.22 (−1.10 to 1.54)	0.73 (−0.78 to 2.23)
Day 42 of 1st round	−0.27 (−1.69 to 1.15)	0.07 (−1.33 to 1.47)	0.69 (−0.56 to 1.94)	1.19 (−0.07 to 2.44)
Follow-up				
6 months	1.14 (−0.68 to 2.95)	0.62 (−1.13 to 2.37)	0.41 (−1.06 to 1.88)	0.33 (−1.15 to 1.81)
12 months	0.48 (−0.81 to 1.77)	0.24 (−0.97 to 1.46)	−0.29 (−1.24 to 0.66)	−0.28 (−1.26 to 0.69)
18 months	0.73 (−0.90 to 2.36)	0.81 (−0.70 to 2.31)	−0.30 (−1.50 to 0.89)	−0.34 (−1.52 to 0.85)
24 months	−0.27 (−1.47 to 0.94)	−0.68 (−1.76 to 0.39)	0.14 (−0.71 to 1.00)	0.32 (−0.52 to 1.16)
36 months	0.08 (−1.38 to 1.54)	−0.72 (−1.91 to 0.48)	−0.37 (−1.36 to 0.63)	−0.44 (−1.43 to 0.56)
60 months	−0.17 (−2.19 to 1.85)	0.01 (−1.71 to 1.73)	−0.34 (−1.72 to 1.05)	0.15 (−1.25 to 1.55)

a Adjusted for clinical center, treatment group, race, sex, parents’ education, single parent, age at test, height, and BMI.

* *p* < 0.05.
